# Intraosseous myofibroma mimicking an odontogenic lesion: case report, literature review, and differential diagnosis

**DOI:** 10.1186/s12957-024-03520-4

**Published:** 2024-09-12

**Authors:** José Wittor de Macêdo Santos, Benito K. Benitez, Daniel Baumhoer, Daphne Schönegg, Thomas Schrepfer, Andreas. A. Mueller, Florian M. Thieringer

**Affiliations:** 1 Oral and Craniomaxillofacial Surgery, University Hospital Basel, Spitalstrasse 21, Basel, 4031 Switzerland; 2 Pediatric Maxillofacial Surgery, University Children’s Hospital Basel, Spitalstrasse 33, Basel, 4031 Switzerland; 3 Bone Tumor Reference Center, Basel Research Centre for Child Health, Institute of Medical Genetics and Pathology, University Hospital Basel, Spitalstrasse 21, Basel, CH-4031 Switzerland; 4 Department of Otolaryngology, University of Florida, 1345 Center Drive, Box 100264, Gainesville, Florida, 32610 USA

**Keywords:** Myofibroma, Oral surgery, Oral Pathology, Pediatrics, Case report

## Abstract

**Background:**

Intraosseous myofibroma of the jaw is a rare neoplasm of mesenchymal origin with limited comprehensive understanding. It typically affects patients in the first two decades of life with a male predilection.

**Case presentation:**

This study presents a rare case of myofibroma mimicking an odontogenic lesion in a 2-year-old boy. The patient presented with an incidental finding of a painless swelling of the right mandibular ramus of unknown etiology. Imaging analysis revealed a solid, expansile lesion adjacent to the germinal zone of the right mandibular first molar. Histopathologic analysis and immunohistochemistry after incisional biopsy suggested a possible central odontogenic fibroma, and the patient underwent total enucleation, leading to the final diagnosis of intraosseous myofibroma. Follow-up examinations showed no evidence of recurrence.

**Conclusions:**

This report contributes to the understanding of myofibroma in pediatric patients and underscores the critical role of meticulous histopathologic examination for effective surgical planning and optimal patient outcomes.

## Introduction

Myofibroma is a rare tumor with a predilection for the head and neck region. Its intraosseous variant stands out as a rare spindle cell tumor involving the jaws, with only a few well-described case series documented in the literature [1]. Typically, intraosseous myofibroma affects patients in the first two decades of life and is more common in males [1–4]. Clinically, these lesions generally present as an asymptomatic pink to red and firm mass in the oral region. Radiographically, unilocular and well-circumscribed osteolysis with potential tooth displacement and/or bone expansion may be seen [1, 3].

Histologically, myofibroma is characterized by a polylobulated spindle cell proliferation arranged in a biphasic pattern. The tumor cells stain positive for alpha-smooth muscle actin (α-SMA) and are usually negative for myogenin, desmin, CD34, S-100 protein, and beta-catenin [5]. This study describes a case of a mandibular intraosseous myofibroma in a 2-year-old boy mimicking an odontogenic tumor and discusses the differential diagnoses and treatment considerations.

## Case report

This report follows the CARE [6] guideline. A 2-year-old boy presented with his parents for a routine pediatric examination, during which a previously unnoticed swelling of the right cheek was noted. The patient had no symptoms, and his medical and family history were unremarkable. Ultrasonography and subsequent magnetic resonance imaging (MRI) were performed, and the patient was referred to our tertiary center for further evaluation.

**Fig. 1:**
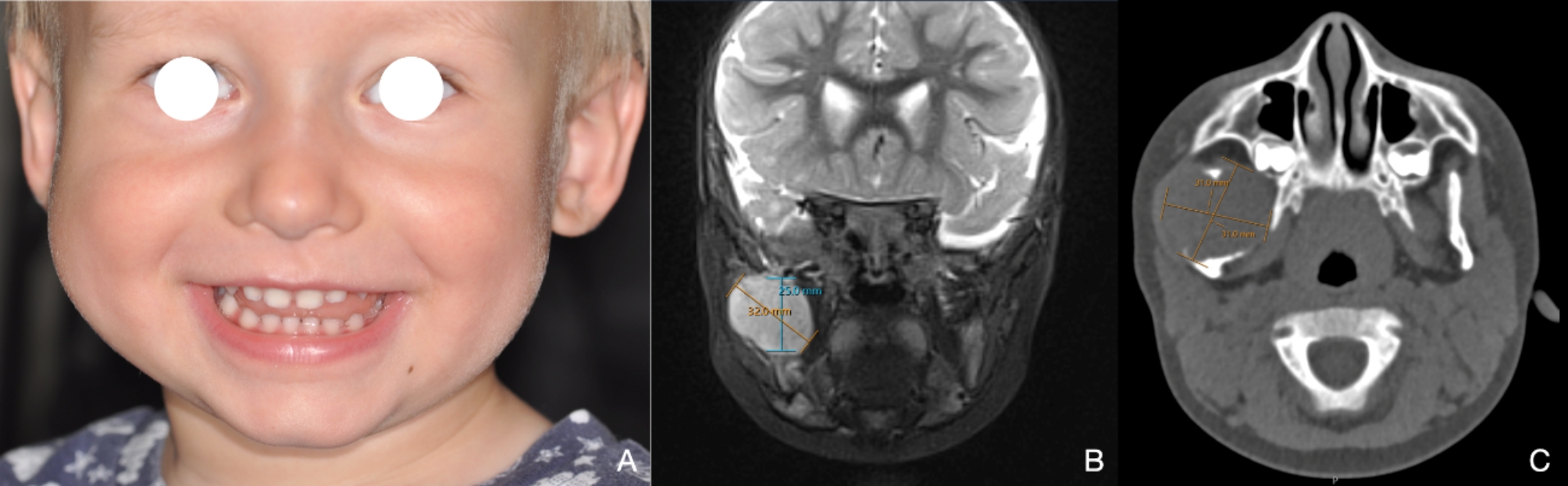
**A**, Clinical frontal view at consultation with volume plus on the right side. **B**, MRI showing a well-delineated intraosseous contrast-enhancing mass along the right mandibular ramus and coronoid process without signs of infiltrative growth or neck involvement. **C**, CT highlighting the lesion at the full height of the coronoid process, extending medially into the pterygomaxillary and laterally into the masticator space

The patient presented with a round, firm swelling in the region of the right mandibular ramus without associated pain, dysfunction, paresthesia, or palsy (Fig. 1A). The previous MRI showed a well-defined intraosseous contrast-enhancing mass along the right mandibular ramus without unequivocal infiltrative growth or signs of an aggressive periosteal reaction, measuring 3.2 × 2.9 × 2.5 cm (Fig. 1B**)**. No additional manifestations were found on clinical and radiological examination, excluding myofibromatosis. A computed tomography (CT) was recommended to delineate the extent of the lesion before any intervention. The lesion was found at the full height of the coronoid process, extending medially into the pterygomaxillary and laterally into the masticator space (Fig. 1C).

**Fig. 2:**
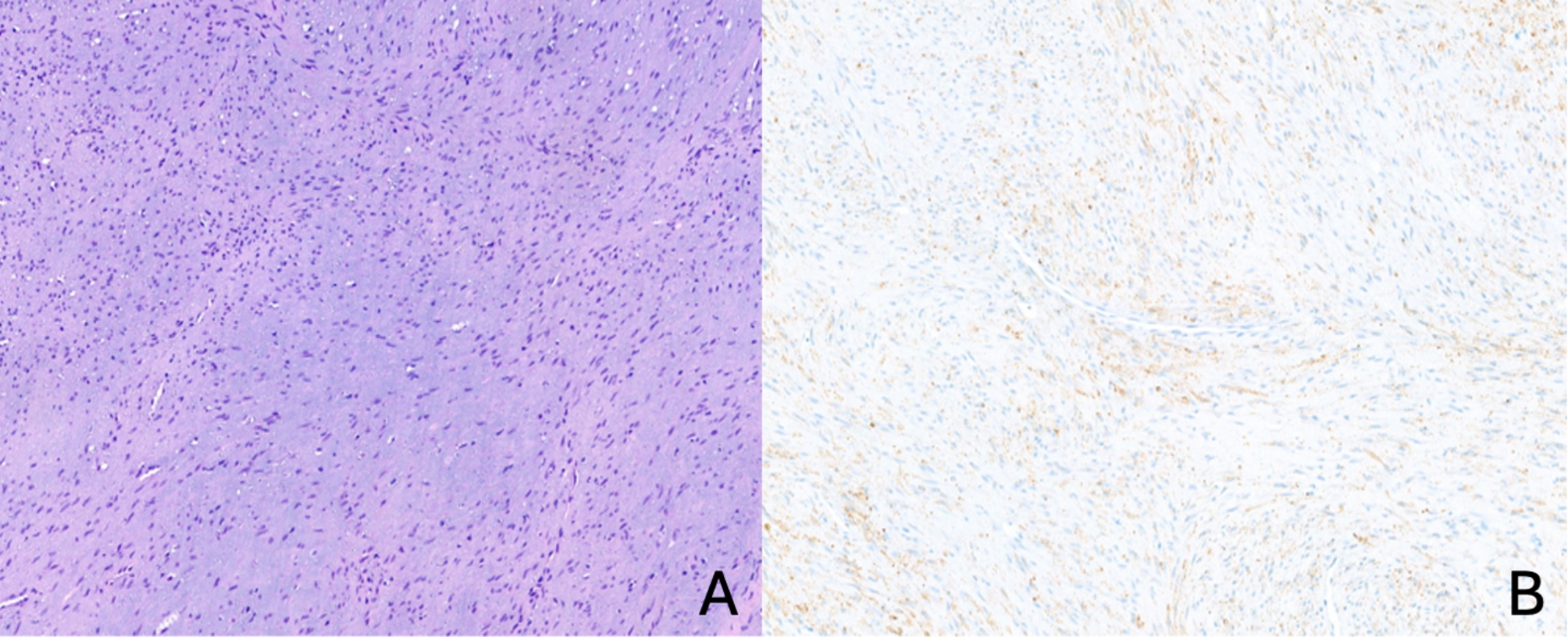
Incisional biopsy (hematoxylin/eosin stain and immunohistochemistry). **A**, monomorphic spindle cell proliferation against a myxoid background and a well-vascularized stroma (**10x**). **B**, weakly positive reaction for α -SMA (**10x**)

After interdisciplinary board discussion, the differential diagnosis included odontogenic tumors, unicystic ameloblastoma, and central giant cell granuloma. Intraoral incisional biopsy under general anesthesia and complete laboratory evaluation were recommended. Laboratory tests were normal. Histopathologic assessment revealed a monomorphic and well-vascularized lesion with a myxoid background. Immunohistochemical studies showed a low proliferation index (Ki-67 positive in 5–7% of the lesional cells). The stainings against pan-CK (CK22), CD31, PU.1, CD45, ERG, beta-catenin, desmin, and MyoD1 yielded negative results. Weak positivity for α-SMA and nestin was noted. The overall constellation was interpreted as being most likely in keeping with a benign mesenchymal odontogenic tumor, e.g., odontogenic fibroma or fibromyxoma, excluding the former non-odontogenic differential diagnoses (Fig. 2A-B).

**Fig. 3:**
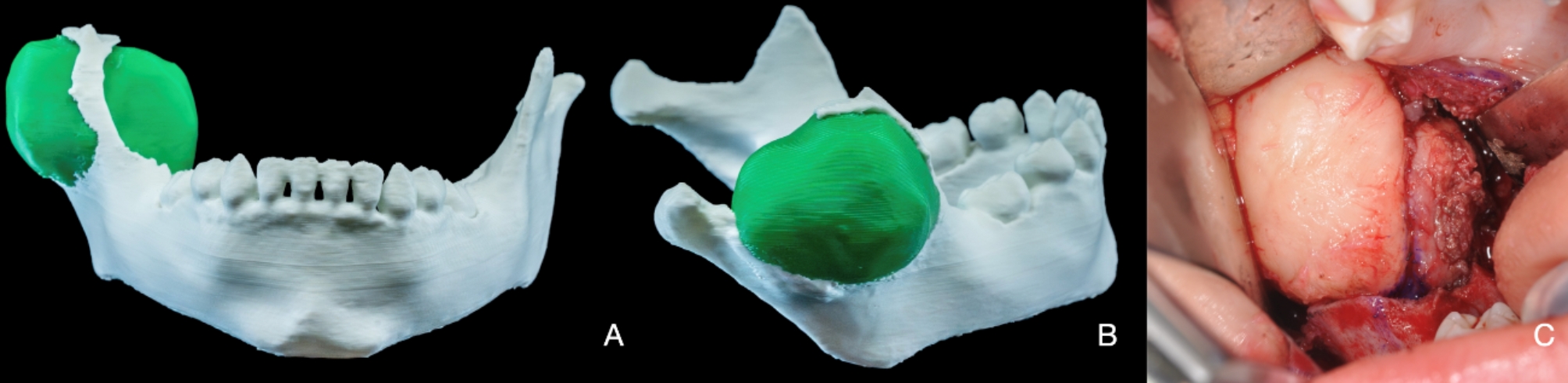
Anatomical 3D-printed model and intra-operative picture. **A-B**, 3D-printed mandible model with a segmented lesion in green. **C**, well-demarcated lesion after retromolar intraoral access

Considering the age and a favorable prognosis, an intraoral enucleation under general anesthesia was planned. Radiology data was segmented to create an anatomical 3D-printed model (Fig. 3A-B**).** Surgery was executed by a senior experienced maxillofacial surgeon without complications, with preservation of the inferior alveolar nerve and no need for bony reconstruction (Fig. 3C).

**Fig. 4:**
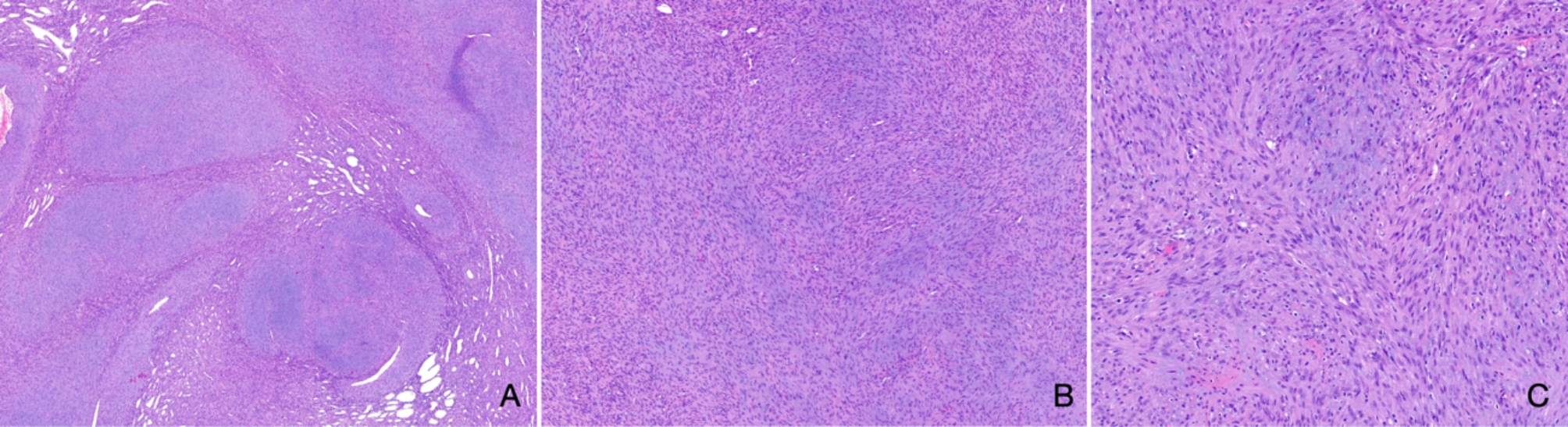
Resection specimen (hematoxylin & eosin staining). **A**, lobulated and biphasic spindle cell proliferation separated by fibrous septae with increased capillary density (**1.6x**). **B-C**, the background appears slightly myxoid, the cells are monomorphic and lack higher-grade cytologic atypia **(5.2x**,** 10x**)

**Fig. 5:**
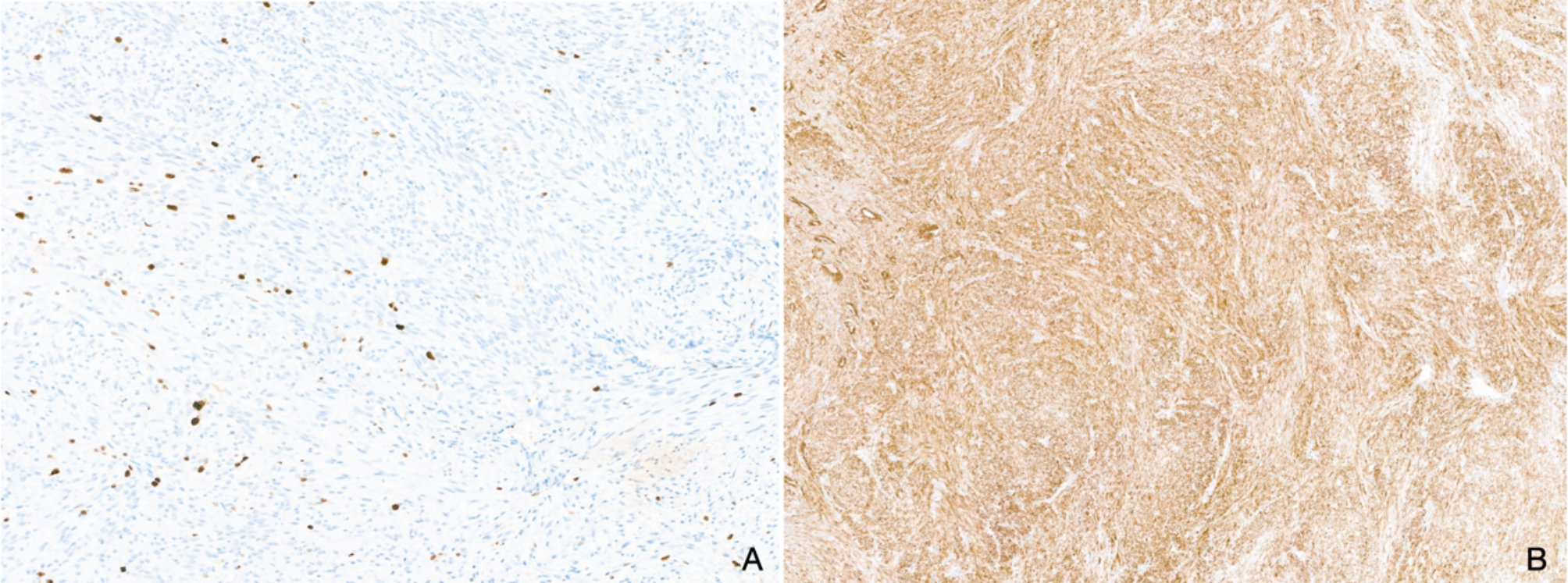
Resection specimen (immunohistochemistry). **A**, the proliferation marker Ki-67 stains 5–10% of tumor cells (**10x**). **B**, the reaction against α-SMA is strongly and consistently positive (**5.2x**)

Histopathological evaluation after marginal excision revealed a monomorphic and partly fascicular spindle cell proliferation with a partly myxoid background (Fig. 4A-C). The complete specimen allowed us to assess better the tumor’s architecture, which showed a lobulated and biphasic morphology with alternating hypo- and hypercellular areas and a densely vascularized background. Immunohistochemistry against α-SMA turned out consistently and strongly positive, whereas the cells remained negative in stainings against calponin, desmin, MyoD1, myogenin, CK22, CK8/18, and S100. The Ki-67 index stained 5–10% of tumor cells. Morphology, immunophenotype, and site were interpreted as diagnostic for an intraosseous myofibroma (Fig. 5).

**Fig. 6:**
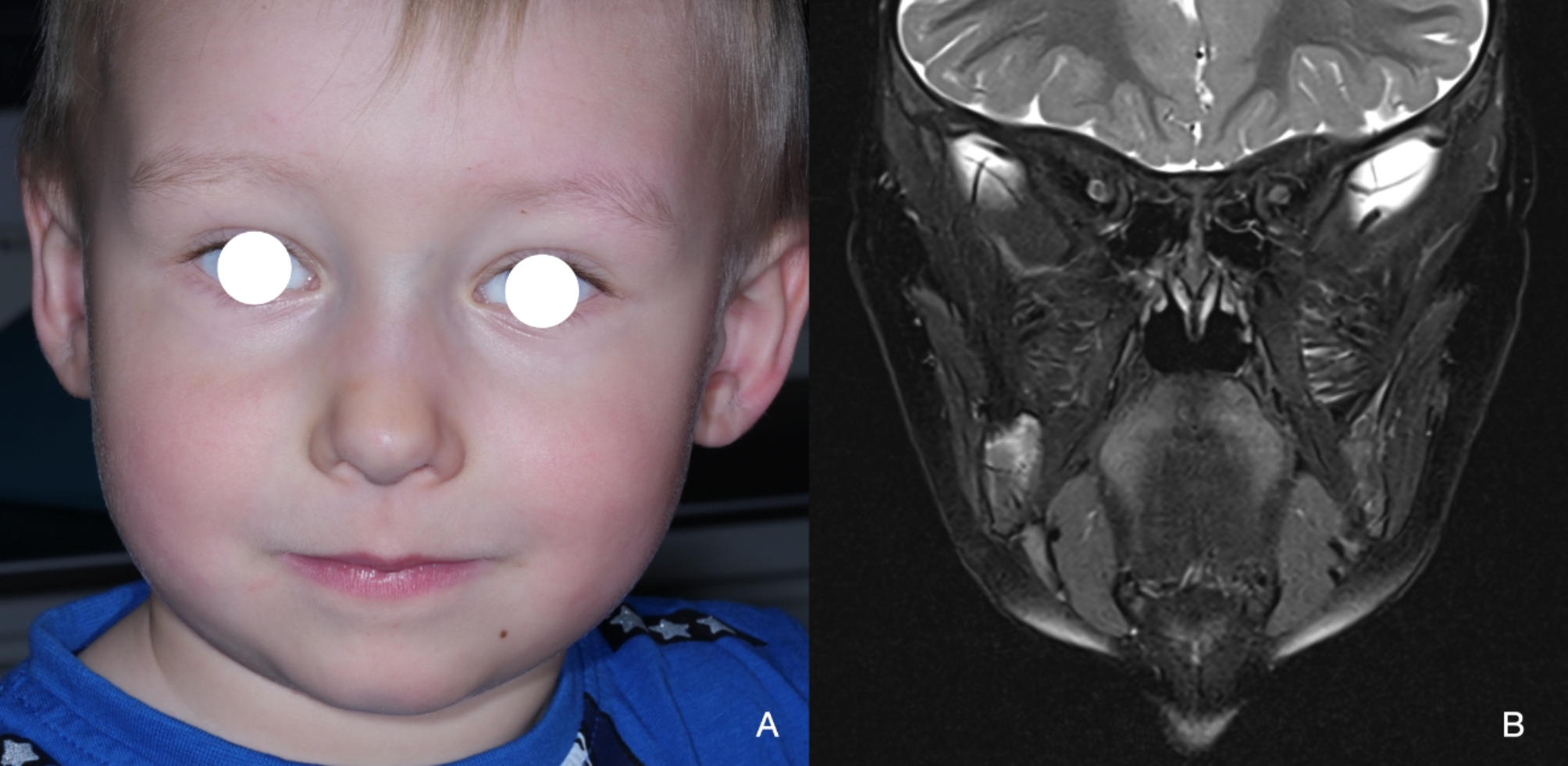
Post-operative follow-up at six months. **A**, clinical aspect with resolution of facial asymmetry at cheek level. **B**, MRI showing no recurrence or tumor persistence but signs of bone remodeling

The patient has been under follow-up for 12 months, demonstrating a normal mouth opening without complaints, palsy, or paresthesia (Fig. 6A**)**. A post-operative MRI conducted six months after surgery revealed no evidence of local recurrence or tumor persistence, a regular appearance of the joint, normal adjacent soft tissues, and the expected amount of bone remodeling (Fig. 6B**)**. These findings underscore the effectiveness of the treatment and point to a favorable prognosis for the patient.

The parents and the surgeon expressed satisfaction with the treatment, considering it well-tolerated by the patient and with aesthetic and functional maintenance. Since the successful resolution was achieved through enucleation only, diligent follow-ups remain essential.

## Discussion

Myofibroma is a rare benign spindle cell tumor that can occur in both soft tissue and bone in children [2]. Solitary and multicentric forms can be distinguished, although some authors consider solitary myofibroma a variant of myofibromatosis. In contrast, others believe it is a separate lesion, with the most recent classification considering it under the category of myopericytomas / perivascular tumors [1]. Maxillofacial lesions are rare, corresponding to 2% of all cases, and usually affect the soft tissues [3]. Intraosseous myofibroma of the jaw is considered rare to ultra-rare and has a preference for the mandible [3, 7]. The age range of patients with myofibroma varies between studies from birth to older age. However, the intraosseous variant of myofibroma is characteristically seen in the first two decades of life, especially before the age of two. Therefore, an intraosseous lesion in this patient population should include intraosseous myofibroma in the differential diagnosis [1–3]. Although this also varies widely between studies, males are more likely to be affected, ranging from 1.2:1 to 2.3:1 ^1,2,3,4^. The clinical aspects of the present case are well in line with those of previously reported cases: painless swelling without any other symptoms. Myofibromas may also present with intraoral growth, ulceration, as well as a rapid size increase, and mimic a malignant tumor [1–8].

Radiographically, intraosseous myofibroma are well-defined, unilocular, and radiolucent lesions that may or may not be associated with an unerupted tooth [1, 3, 4]. They can exhibit a multilocular aspect, tooth resorption or displacement, cortical bone expansion, or cortical perforation [1, 4]. The present case was classic in presentation but was already large and close to the condyle with some bone expansion and cortical perforation. Since the patient’s parents did not notice the lesion before the pediatricians’ inspection, estimation of the growth dynamics was not possible.

Definitive diagnosis is usually difficult due to the rarity of the lesion, the equivocal clinical, radiographic, and histological findings as well as the lack of specific biomarkers. Myofibroma is therefore prone to misdiagnosis with the consequent risk of overtreatment [1, 4, 6, 7]. In the case presented here, the initial biopsy showed a non-specific spindle cell proliferation with only weak α-SMA staining, pointing to the differential diagnoses of odontogenic fibroma or fibromyxoma. Only the resection specimen showed the typical polylobulated biphasic architecture and stronger staining for α-SMA, allowing the correct diagnosis of myofibroma. Incisional biopsies must always be interpreted cautiously and can sometimes be misleading, particularly if dealing with lesions for which specific biomarkers are not available [1, 9]. In the literature, even cases initially interpreted as leiomyosarcomas have been reported, resulting in overtreatment of the patient with *en bloc* resection [10].

Regarding the differential diagnosis before incisional biopsy, odontogenic lesions such as odontogenic keratocysts (OKCs) and unicystic ameloblastoma were considered. OKCs typically originate in tooth-bearing regions, with a high prevalence in the posterior mandible, particularly among males, as in the present case [11]. However, several distinct features differentiate OKCs from myofibromas: the age range of involvement (8–82 years, with a peak in the third decade of life), the mesiodistal growth pattern rarely exhibiting cortical expansion, and the absence of a cystic lining covered by the pathognomonic basal layer with hyperchromatic cells in palisade [11].

Although unicystic ameloblastoma is considered in the differential diagnosis because of its location and radiographic similarities to solitary intraosseous myofibroma, it is most commonly seen in the third to sixth decades of life and is much less common than its multicystic counterpart [12]. In addition, our findings after an incisional biopsy, a well-vascularized solid lesion with monomorphic spindle cell proliferation on a myxoid background, suggested a mesenchymal tumor, thereby narrowing our differential hypotheses. In contrast, unicystic ameloblastomas typically present with an epithelial lining composed of loosely cohesive cells and a basal layer of columnar or cuboidal cells with hyperchromatic nuclei, reverse polarity, and basilar cytoplasmic vacuolization, resembling the stellate reticulum [12].

Beyond odontogenic lesions, other entities that pose a greater challenge in differentiating from myofibromas include desmoplastic fibromas [3]. This rare fibroblastic tumor is often considered as the intraosseous counterpart of desmoid fibromatosis [4]. Similar to our case, desmoplastic fibromas primarily affect young patients, typically manifesting as a painless swelling in the posterior mandible, though with more aggressive and destructive behavior [4, 13]. Additionally, its occasional unilocular radiolucent appearance, combined with a similar histopathological spindle-cell fascicular architecture, can mimic myofibromas [4]. However, the absence of a hemangiopericytoma-like vascular pattern and less consistent α-SMA positivity in desmoplastic fibromas, as opposed to the pattern and strong α-SMA positivity in myofibromas, helps in distinguishing between the two lesions [14, 15].

The rarity of well-documented cases with long-term follow-up and the limited experience with these intraosseous lesions, in general, make it difficult to predict these lesions’ behavior and the optimal treatment/follow-up. The typical characteristics of well-defined borders, benign nature, and low recurrence rates make conservative surgery with enucleation with/without curettage the treatment of choice [7]. In addition, wide excision and extraction of associated teeth are alternative approaches [1]. Instead of a specific protocol, it has been suggested to treat benign pediatric jaw tumors according to their biological behavior, with aggressive tumors requiring resection and nonaggressive tumors requiring enucleation [10].

A minimally invasive approach seems crucial when dealing with solitary myofibroma in pediatric patients to avoid lifelong sequelae. The short follow-up is a limitation of the present report. Nevertheless, we present a rare and well-documented case, including 3D planning, and discuss specific histologic features with differential diagnosis after rigorous investigation.

## Conclusions

Like other rare pediatric mass lesions, intraosseous myofibroma presents a diagnostic challenge due to its non-specific histopathological features, particularly in biopsy material. In addition, there are no pathognomonic molecular markers. Early recognition and correct diagnosis are essential for effective management, as misdiagnosis may be associated with overtreatment and morbidity. This report describes a well-documented case of a rare disease for which diagnosis and treatment can be challenging. It highlights the importance of meticulous histopathological examination for well-planned surgery to ensure optimal outcomes and minimal morbidity.

## Data Availability

No datasets were generated or analysed during the current study.

## Abbreviations

α-SMA: alpha-smooth muscle actin
MRI: magnetic resonance imaging
CT: computed tomography
OKCs: odontogenic keratocysts
